# The New Union among Matrons

**Published:** 1919-05-17

**Authors:** 


### THE MATRONS' AND SISTERS' DEPARTMENT.

### THE NEW UNION AMONG MATRONS.

The desire for a closer rapprochement among matrons of institutions for the sick has long been felt, and recent events have accentuated it. Accordingly it is not surprising that with almost incredible swiftness an Association of Hospital Matrons has been formed and that some eighty-eight matrons from all parts of the kingdom found it possible to gather at St. Thomas's on the invitation of Miss Lloyd Still on May 8, in order to assist at the birth of the new society. The inauguration was timed auspiciously. Momentous decisions have to be taken within the next week or two. And none have greater authority to speak for the nursing profession than these heads of nursing departments, whose fitness to organise was tested for many years before they reached their present positions of authority, and whose experience of nursing under all its aspects is profound and unbiased. We hail with the greatest satisfaction this new proof of the progressive character of the modern nursing profession. In former times matrons were too apt to allow their horizon to be bounded by their hospital walls. They shrank from discussion and held aloof from one another. That they have awakened to the perception that there is much awaiting accomplishment which cannot be done in isolation marks a new spirit and foretells the coming of a new force. In this the College of Nursing has been the forerunner.

The attempt to breed dissension between matrons and nurses has so conspicuously failed that nurses need to be adjured to elect at least a decent proportion of nurses who are not matrons on the Council of the College. We have never believed in the theory that matrons' interests differed from those of other members of the profession. Holding office is often but a very arduous episode in the life of the nurse, and it is ridiculous to suppose that she changes her ideals because she is responsible for running the machine. But even a brief experience of authority does enlarge the boundaries of a woman's knowledge and teach her much about her profession which years of private or institutional nursing had failed to bring to her notice. A woman who has experienced some of the cares and used some of the opportunities which office brings can hardly fail to be in a better position to form a judgment on nursing matters than one whose attention has always been concentrated merely on her own job. Meeting in council together matrons have something to get and something to give. Their unanimous conclusions are entitled to the respect of the profession and of the public.

The need for uniting authoritative nursing opinion is urgent. Our readers will have noticed in our last issue that a Bill to provide for the training and registration of nurses was introduced into the House of Lords on May 1 by Viscount Goschen, that it was read a first time and has been printed and circulated. The objectionable features of the Registration Bill now before the House of Commons were not, as it was hoped might be the case, removed during its consideration by the Standing Committee. That Committee, in fact, took so small an interest in what concerned the nurses' future, that out of some sixty-five members sufficient were not forthcoming to form a quorum on an occasion when vital alterations were to be discussed. The passage of this Bill in its present form would prove an irretrievable calamity for the nursing profession, for it would be handed over shackled to the domination of persons antagonistic to modern nurses and to their representatives. It will be opposed and being opposed will not become law.

The alternative to the postponement of registration for an indefinite time lies in the Bill now before the House of Lords. It is the Bill drawn up by the College of Nursing, and with its provisions all are familiar. The main principles of the Bill are approved by an immense majority of those to whom the nation owes its present supply of nurses. It is approved in principle, we venture to assert, though there may be one or two exceptions, by the nurse-training schools of the whole Kingdom. The new Association of Matrons has come together at the psychological moment, when the matrons' enthusiastic adherence to the measure before the House of Lords undoubtedly must contribute largely to its welcome and acceptance.

